# Adipotropic effects of heavy metals and their potential role in obesity

**DOI:** 10.12703/r/10-32

**Published:** 2021-03-26

**Authors:** Alexey A Tinkov, Michael Aschner, Tao Ke, Beatriz Ferrer, Ji-Chang Zhou, Jung-Su Chang, Abel Santamaría, Jane C.-J. Chao, Jan Aaseth, Anatoly V Skalny

**Affiliations:** 1 IM Sechenov First Moscow State Medical University (Sechenov University), Moscow, Russia; 2 Yaroslavl State University, Yaroslavl, Russia; 3 Department of Molecular Pharmacology, Albert Einstein College of Medicine, Bronx, NY, USA; 4 School of Public Health (Shenzhen), Sun Yat-sen University, Shenzhen, China; 5 Taipei Medical University, Taipei, Taiwan; 6 Laboratorio de Aminoácidos Excitadores, Instituto Nacional de Neurología y Neurocirugía, S.S.A., Mexico City, Mexico; 7 Nutrition Research Center, Taipei Medical University Hospital, Taipei, Taiwan; 8 Research Department, Innlandet Hospital Trust, Brumunddal, Norway

**Keywords:** mercury, cadmium, lead, adipogenesis, adipocyte

## Abstract

Epidemiological studies demonstrated an association between heavy metal exposure and the incidence of obesity and metabolic syndrome. However, the particular effects of metal toxicity on adipose tissue functioning are unclear. Therefore, recent findings of direct influence of heavy metals (mercury, cadmium, and lead) and metalloid (arsenic) on adipose tissue physiology are discussed while considering existing gaps and contradictions. Here, we provide a literature review addressing adipose tissue as a potential target of heavy metal toxicity. Experimental *in vivo* studies demonstrated a significant influence of mercury, cadmium, lead, and arsenic exposure on body adiposity. In turn, *in vitro* experiments revealed both up- and downregulation of adipogenesis associated with aberrant expression of key adipogenic pathways, namely CCAAT/enhancer-binding protein (C/EBP) and peroxisome proliferator-activated receptor gamma (PPARγ). Comparison of the existing studies on the basis of dose and route of exposure demonstrated that the effects of heavy metal exposure on adipose tissue may be dose-dependent, varying from increased adipogenesis at low-dose exposure to inhibition of adipose tissue differentiation at higher doses. However, direct dose-response data are available in a single study only for arsenic. Nonetheless, both types of these effects, irrespective of their directionality, contribute significantly to metabolic disturbances due to dysregulated adipogenesis. Particularly, inhibition of adipocyte differentiation is known to reduce lipid-storage capacity of adipose tissue, leading to ectopic lipid accumulation. In contrast, metal-associated stimulation of adipogenesis may result in increased adipose tissue accumulation and obesity. However, further studies are required to reveal the particular dose- and species-dependent effects of heavy metal exposure on adipogenesis and adipose tissue functioning.

## Introduction

Adipose tissue is a metabolically active tissue that evolutionarily developed as a specialized lipid depot also capable of secreting a wide range of signaling molecules (adipokines)^1^. Perturbations in adipose tissue physiology are known to be implicated in a wide range of metabolic disturbances, and obesity is the most widespread^2^. Findings in the last decades demonstrated that exposure to environmental pollutants may play a significant role in the development of obesity because of their role as endocrine disruptors, raising the concept of “obesogens”^3^. Known chemical obesogens include phenols, polycyclic aromatic hydrocarbons, amides, metallic compounds, esters, halogenated compounds, air pollutants, and flavoring agents, to name a few^4^.

Specifically, both epidemiological and experimental studies demonstrated an association between persistent organic pollutant exposure and pathogenesis of obesity and this was due mainly to interference with adipogenesis through modulation of peroxisome proliferator-activated receptor gamma (PPARγ) and CCAAT/enhancer-binding protein (C/EBP) expression. Certain obesogens—organotin compounds, in particular—are considered agonists of nuclear receptors (PPARγ and RXR). Transgenerational effects of obesogens may also be mediated through epigenetic mechanisms, including altered DNA and histone methylation, as well as impaired chromatin structure^4^.

Recent findings demonstrated the association between heavy metal exposure and prevalence of obesity. Exposure to markers of mercury^5^, cadmium^6^, lead^7^, and arsenic^8^ as well as metal mixture^9^ were found to be correlated with anthropometric and metabolic parameters in obesity and metabolic syndrome. However, the only metal considered a classic obesogen is tin (Sn), particularly its organic compounds, organotins^10^, which, being both PPARγ and RXR agonists, were shown to interfere with adipogenesis^11^. Data on the impact of other metals on adipogenesis and adipocyte functions are extremely insufficient. Specifically, a crude PubMed-Medline search using the terms “adipocyte” and “cadmium” or “mercury” revealed 21 and 13 papers, compared with 52, (48) 193, and 77 for “organotin”, “(polychlorinated) biphenyl”, and “dioxin”, respectively, indicating a scant number of studies on the adipocyte-targeted effects of heavy metals. Therefore, we aimed to discuss recent findings on the direct influence of heavy metals (Hg, Cd, and Pb) and metalloid (As) on adipose tissue physiology in view of the existing gaps and contradictions.

## A brief introduction to the role of adipose tissue in the regulation of body adiposity

The cardinal morphological feature of obesity is increased white adipose tissue (WAT) mass (adiposity) that results from the imbalance between energy intake and energy expenditure. At positive caloric balance, excessive energy is stored as lipids in adipocyte lipid droplets as a result of lipogenesis, whereas increased energy expenditure activates mobilization of the stored lipids through lipolysis, which is regulated by neuroendocrine signals^12^. In parallel to the changes in adipocyte size, which are dependent on the balance between lipogenic and lipolytic enzymes, adipose tissue remodeling is also associated with modulation of adipogenesis^13^. Adipogenesis is the process of proliferation and subsequent maturation of adipocytes from adipocyte progenitors regulated by C/EBPs and PPARγ to meet the increasing lipid-storing requirements. In turn, adipogenesis dysregulation was shown to be associated with altered metabolic profile in obesity^14^. In addition, owing to the role of adipose tissue as an endocrine organ, adipocyte dysfunction results in impaired adipokine secretion^15^. Leptin, the main endocrine product of adipose tissue, is known to play a significant role in the central control of energy balance by influencing hypothalamic centers of appetite and satiety, also underlining the role of adipose tissue in the regulation of energy intake. Therefore, altered leptin signaling is known to be associated with impaired feeding behavior and obesity^16^. It is also notable that under certain signals WAT may modulate energy expenditure through modulation of beige adipogenesis and white-to-beige adipocyte transformation and subsequent beige thermogenesis. In turn, beige-to-white adipocyte transformation may further aggravate obesity^17^. Therefore, the role of adipose tissue is mediated not only by its role as lipid reservoir but also through modulation of adipogenesis, adipokine secretion, involvement in the central regulation of appetite and satiety as well as the capability to regulate thermogenesis via beige (“brite”) adipocyte formation^18^.

## Adipotropic effects of heavy metals

### Mercury (Hg)

High total blood Hg levels were found to be associated with significantly increased visceral adipose tissue mass in Korean adults^19^. Experimental studies also demonstrated the impact of Hg exposure on adipose tissue accumulation. Specifically, periconceptional maternal exposure to methylmercury (MeHg) and cadmium chloride (CdCl_2_) (both 2 mg/kg) resulted in increased adipose tissue mass and body weight in offspring with transgenerational effect that persisted to the F4 generation^20^.

Rizzetti *et al*. (2019) demonstrated that mercury chloride (HgCl_2_), when injected intramuscularly (4.6 μg/kg as the first dose with subsequent 0.07 μg/kg per day exposure for 60 days) to male Wistar rats, may be considered a “powerful environmental WAT disruptor” that is capable of reducing adipocyte size^21^. The latter was shown to be accompanied by increased adiponectin, leptin, PPARα, and PPARγ mRNA expression, indicative of impaired adipogenesis and adipokine secretion. The revealed increase in adipose tissue GRP78, CHOP, and CD11 mRNA expression indicated the potential role of endoplasmic reticulum stress and pro-inflammatory signaling in Hg-induced alteration of adipose tissue physiology^21^. These findings only partially corroborate the pioneering observation by Kawakami *et al*. (2012)^22^, who also demonstrated Hg-induced decrease in adipose tissue mass and adipocyte size when high-fat diet (HFD)-fed male Slc:ICR mice were subcutaneously injected with 1.0 mg/kg body weight HgCl_2_. However, inorganic mercury exposure in HFD-fed animals significantly reduced WAT-specific leptin, PPARα, and PPARγ mRNA expression that may be at least partially mediated by Hg-induced AMPK upregulation^22^. Certain inconsistencies between the adipotropic effects of Hg reported in these two studies may be explained by the difference in the dose of metal exposure; higher dose^22^ results in adipogenic response inhibition due to the potential toxic (including pro-oxidant) effects. In addition to the studies in mice demonstrating a significant impact of Hg exposure on PPARγ, our recent findings from a *Caenorhabditis elegans* model revealed a significant impact of 10 to 20 µM MeHg on lipid metabolism regulatory genes, including pro-adipogenic worm orthologs to human SREBP and C/EBPs^23^, due to MeHg being another regulator of adipogenesis. In contrast to the previously mentioned studies, one study demonstrated that in C57BL/6J mice orally exposed to 0.5 or 5 ppm MeHg (concentrations that failed to induce adipogenic gene expression in visceral adipose tissue), a reduction of adipose tissue cumulation was associated with MeHg-induced increase in hypothalamic pro-opiomelanocortin (POMC) expression^24^. Given the role of POMC as an anorexigenic peptide that reduces food intake^25^, the obtained data are indicative of the role of central effects of Hg in the modulation of body adiposity^24^.

The role of Hg as a potential factor affecting adipogenesis was also demonstrated in *in vitro* studies. The most recent study demonstrated that MeHg exposure *in vitro* (0, 0.3, 1.7, or 3.8 mM) for 6 days increased lipid accumulation in 3T3-L1 preadipocytes isolated from perivisceral adipose tissue. Although these changes were accompanied by an increase in fatty acid synthase and perilipin expression, certain other adipogenic markers, namely fatty acid transport protein 1, glycerol-3-phosphate dehydrogenase, and C/EBPδ, were downregulated^26^. It is also notable that Hg is capable of regulating adipogenesis-related genes, including *C/EBPβ*, *DDIT3* (both upregulation), *LPIN1*, and *SREBF1* (both downregulation), in BEAS-2B cells when administered together with 2,3,7,8-tetrachlorodibenzo-p-dioxin (TCDD)^27^.

The observed effects of Hg on adipogenesis also corroborated *in vitro* data on its impact on adipokine secretion. Specifically, treatment of 3T3-L1 adipocytes to 200 ng/mL MeHg resulted in the formation of a lower number of adipocytes and clumped lipid droplets as well as activation of apoptosis through induction of oxidative stress as evaluated by increased culture 4-hydroxynonenal (4-HNE) levels. These changes were accompanied by aberrant adipokine expression patterns characterized predominantly by elevated adiponectin and resistin production^28^. In addition, MeHg exposure (100–200 ng/mL) resulted in a significant increase in vascular endothelial growth factor (VEGF) production, which is known to play a certain role in the pathogenesis of metabolic syndrome^29^, in mature 3T3-L1 adipocytes^30^. 

### Lead (Pb)

Pb effectively accumulates in human adipose tissue^31^, although direct effects of Pb exposure on adipocyte physiology have yet to be discerned. Lifetime Pb exposure (200–500 ppm) in C57Bl/6 mice** resulted in a significant increase in bone marrow adiposity characterized by increased adipocyte size and number through upregulation of PPARγ gene expression^32^, indicative of Pb-induced adipogenesis. The results of *in vitro* studies have also demonstrated a significant effect of Pb on adipogenesis through modulation of key regulators (PPARγ and C/EBPβ). Particularly, a study in 3T3-L1 fibroblasts demonstrated that Pb exposure (0–10 µM) increased cytosolic lipid accumulation and perilipin expression occurring during adipogenesis. These effects were mediated by upregulation of C/EBPβ and ERK expression with subsequent activation of PPARγ^33^. These findings corroborate earlier observations on the interactive effects of Pb exposure (2 µM) and high-fat medium on the expression of proadipogenic PPARγ and adipogenic marker fatty acid-binding protein 4 (FABP4) in MC3T3-E1 cells^34^. However, higher concentrations (>10 µM) of Pb were shown to inhibit proliferation of 3T3-L1 fibroblasts^35^ that may be associated with toxic effects of increasing metal concentrations.

It is also notable that Pb exposure may affect central adipokine signaling through downregulating adiponectin receptor 1b and especially leptin receptor gene expression in zebrafish brain^36^. Given the role of altered leptin receptor expression in leptin resistance and obesity^37^, these findings may be indicative of the central role of Pb in alterations of energy homeostasis and excessive adiposity.

### Arsenic (As)

As was considered a potential obesogen affecting normal adipose tissue physiology, although direct data on the impact of As species on adipogenesis and adipocyte functions are inconsistent^38^. Experimental *in vivo* studies demonstrated that As exposure is capable of affecting WAT mass, although the observed effects of As on adipogenesis seem to be dose-dependent. Specifically, exposure to As-containing drinking water (300 μg/L sodium arsenite [NaAsO_2_]) for 9 weeks in male C57BL/6J mice resulted in a significant increase in WAT mass, accompanied by impaired mitochondrial biogenesis and thermogenesis due to modulation of PPARα and PPARγ-specific genes, including *Slc27a2*, *Fabp3*, *Ucp1*, *Acsl5*, *Scd2*, and *Cpt1β*^39^. However, exposure to a higher dose (50 mg/L for 16 weeks) significantly reduced adipose tissue mass in HFD-fed male C57BL/6J mice without alteration of energy expenditure as assessed by indirect calorimetry^40^. The authors propose that the observed “antiobesogenic” effect of As could be attributed to its insulin-sensitizing activity^40^. Of interest, an inverse association between As dose and adipogenic response was reported by Shearer *et al*. (2017)^41^. Particularly, oral exposure to 300 ppb inorganic As in male C57BL/6J mice resulted in a significant decrease in the expression of adipocyte-specific genes in isolated adipose-derived mesenchymal stem/stromal cells (ASCs), whereas ASCs obtained from mice exposed to a higher dose (1000 ppb) were characterized by a significant increase in adiponectin, leptin, and FABP4 expression, indicative of a biphasic response of adipogenesis to inorganic As exposure^41^.

High-dose As exposure was also shown to alter the production of adiponectin. Drinking NaAsO_2_-containing water (5 and 50 ppm in drinking water) for 18 weeks resulted in a significant decrease in serum adiponectin levels in male C57BL/6 mice^42^. However, the impact of As on adiponectin production seems to be diet-dependent. Particularly, As exposure in male mice fed a low- and high-fat diet resulted in a significant decrease and increase of circulating adiponectin levels, respectively^43^. Reduced levels of adiponectin in response to As toxicity may also indirectly indicate inhibitory effects of the metalloid on adipogenesis and production of adipocyte-specific proteins, although this relationship may be mediated by factors such as insulin resistance, inflammation, atherogenic dyslipidemia, and total body adiposity^44^.

The existing *in vitro* studies further unraveled the mechanisms underlying the modulatory effect of As on adipogenesis. Generally, previously discussed *in vivo* studies corroborate earlier data on the inhibitory effect of As (0.2–4 μM) on adipogenic differentiation of mesenchymal stem cells through downregulation of PPARγ and C/EBP expression^45^. The results of a recent study performed in stromal vascular fraction cells isolated from WATs of Nrf1(f)-knockout mice allow investigators to propose that As-induced inhibition of PPARγ signaling and adipogenesis may be NRF1-dependent^46^. Inhibition of PPARγ signaling was shown to occur because of As (5–10 μM)-induced endoplasmic reticulum stress in 3T3-L1 preadipocytes with subsequent induction of C/EBP homologous protein (CHOP10) that is known to reduce C/EBPβ DNA-binding activity^47^. Modulation of SIRT3-FOXO3a, endothelin-1, Ras-MAP-AP-1, and PI3K-Akt pathways by As exposure may also significantly contribute to disturbed adipocyte functioning^48^. As exposure (0 or 100 μg/L in drinking water or 1 μM in culture medium) was also shown to inhibit adipogenesis by increasing miR-29b in human mesenchymal stem cells and murine adipose tissue and sustained cyclin D expression, preventing cell cycle exit and providing a shift from differentiation to proliferation^49^.

Brown adipose tissue (BAT) was also shown to be the potential target of As toxicity mediating the effects of the metalloid on body adiposity. Specifically, oral administration of NaAsO_2_ (5–10 mg/kg) to male C57BL/6J mice was accompanied by a more pronounced As accumulation in BAT as compared with WAT and also resulted in inhibition of brown adipocyte differentiation and decreased expression of PPARγ and other brown adipocyte-specific markers (UCP1 and PGC1). Moreover, As-induced inhibition of autophagy was proposed to play a role in BAT dysfunction^50^. Correspondingly, prolonged exposure of adult C57BL/6J female mice to inorganic As (5–20 ppm in drinking water for 17 weeks) was shown to impair energy homeostasis, resulting in metabolic disturbances attributed to BAT whitening and impaired thermogenesis^51^. These findings corroborate data on As-induced alteration of mitochondrial biogenesis and beige adipocyte formation, indicative of the significant role of reduced thermogenesis in the observed increase in adiposity^40^.

### Cadmium (Cd)

Although the role of Cd exposure in the development of diabetes mellitus type 2 was demonstrated in epidemiological and laboratory studies, the mechanisms of particular involvement of Cd in obesity pathogenesis are unclear^6^. Owing to high abundance in the human body, adipose tissue was considered a significant Cd depot in spite of a rather low Cd level (42 μg/kg)^52^. Correspondingly, Cd levels in adipose tissue were found to be directly associated with smoking^53^, which was the most significant source of non-occupational metal exposure. These findings indicate that adipose tissue may be considered a potential target for Cd toxicity. However, data on the particular effects of Cd in adipose tissue or adipocyte cultures are inconsistent^6^.

Cd levels in prenatal blood were found to be significantly associated with the risk of pediatric obesity in children (4 to 5 years old) living in North Carolina and this is in agreement with the effects of prenatal Cd exposure on juvenile lipid accumulation in zebrafish^54^. Early-life low-dose Cd exposure (100 nM CdCl_2_ with drinking water) was shown to increase body mass, adiposity, and elevated leptin levels in male C57BL/6J mice^55^. It is also notable that periconceptional exposure to Cd and Hg (2 mg/kg each) in CD1 mice caused transgenerational metabolic effects characterized by abdominal obesity and glucose intolerance up to the F4 generation, although the particular impact of combined metal exposure on adipogenesis was not specified^20^. The observed effects of Cd on body adiposity may be mediated by its impact on adipogenesis as demonstrated *in vitro*. Particularly, chronic exposure to 0.5 to 2 µmol/L CdCl_2_ was shown to increase the abundance of bone marrow adipocytes through upregulation of mesenchymal stem cell adipogenic differentiation with concomitant upregulation of PPARγ expression^56^.

However, certain *in vivo* and *in vitro* studies reported opposite effects of Cd exposure on adipose tissue cellularity and function and were also supported by epidemiological data. Particularly, a study originating from Mexico City demonstrated an inverse association between maternal urinary Cd levels and abdominal and peripheric adiposity^57^. An experimental *in vivo* study demonstrated that administration of 100 ppm CdCl_2_ in drinking water to ICR mice for 8 weeks resulted in a significant decrease in adipocyte size but that Cd chelation using dimercaptosuccinic acid (DMSA) ameliorated this effect^58^. This effect may be at least partially mediated by the earlier demonstrated Cd-induced (3 μM) inhibition of adipogenesis and a dose-dependent decrease in C/EBPα and PPARγ protein expression in 3T3-L1 preadipocytes^59^.

The existing experimental studies revealed significant contradictions in the effects of Cd on adiposity and adipogenesis in particular. Hypothetically, “low”-dose exposure *in vivo* (100 nM CdCl_2_ with drinking water, C57BL/6J mice^55^) and *in vitro* (0.5–2 µmol/L CdCl_2_, bone marrow adipocytes^56^) may promote body adiposity through upregulation of adipogenesis, whereas “high” doses studied in ICR mice (100 ppm CdCl_2_ in drinking water^58^) and 3T3-L1 preadipocytes (3 μM^59^) may reduce adipose tissue levels at least partially through inhibition of adipogenesis.

## Conclusion and perspectives 

**Figure 1.**
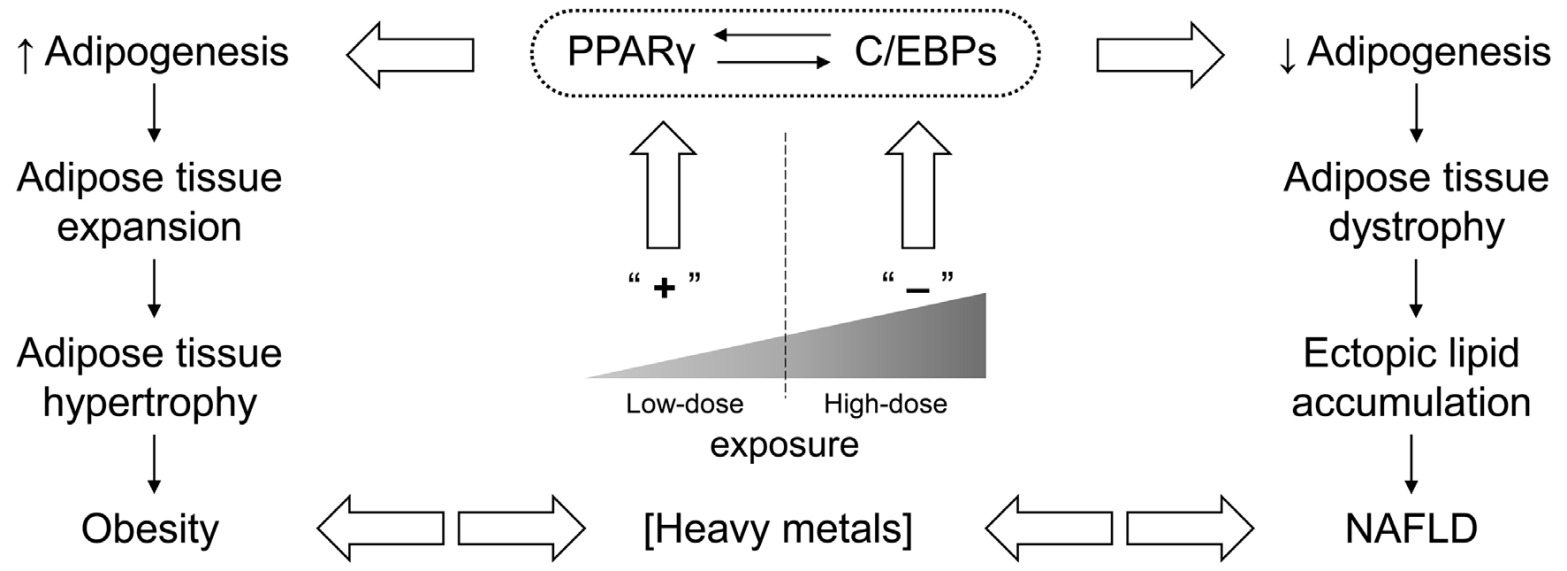
A schematic representation of biphasic adipogenic response to heavy metal exposure. Briefly, low-dose exposure (left) may upregulate key adipogenic factors C/EBPs and PPARγ, thus promoting excessive adipogenesis and contributing to obesity and diabetes mellitus. In turn, “high-dose” metal exposure (right) may inhibit adipogenesis through downregulation of C/EBPs and PPARγ that may be associated with toxic effects of the metals because of pro-inflammatory and pro-oxidant activity. Under positive caloric balance, reduced adipogenic capacity results in increased ectopic lipid accumulation and lipotoxicity, including that in non-alcoholic fatty liver disease (NAFLD). However, *in vivo* and *in vitro* dose-response studies are required to clarify the association between toxic metal exposure and adipogenesis. C/EBP, CCAAT/enhancer-binding protein; PPARγ, peroxisome proliferator-activated receptor gamma.

Generally, the existing *in vivo* and *in vitro* studies demonstrate that heavy metal(loid)s affect adipose tissue mass and function through modulation of adipogenesis (via C/EBPα and PPARγ), indicating the “adipotropic” effects of heavy metals. Despite some existing contradictions, the experimental data provide insight into dose-dependent effects of heavy metals on adipogenesis. It is proposed that the effects of heavy metal exposure on adipose tissue is biphasic (Figure 1), varying from increased adipogenesis at low-dose exposure to inhibition of adipose tissue differentiation at higher doses, as demonstrated for Hg^21,22^, Cd^55,56,58,59^, and As^41^. However, direct dose-response analysis was performed only for the latter^41^. Similar patterns of biological action were shown to underlie the hermetic effect of chemical substances, including heavy metals and metal nanoparticles^60^.

Both stimulation and inhibition of adipogenesis in response to heavy metal exposure might contribute significantly to metabolic disturbances. Particularly, inhibition of adipocyte differentiation is known to reduce lipid-storage capacity of adipose tissue, leading to ectopic lipid accumulation^61^. This assumption is indirectly confirmed by the observed association between heavy metal exposure and non-alcoholic fatty liver disease^62–64^. On the other hand, metal-associated stimulation of adipogenesis may result in increased adipose tissue accumulation and obesity. At the same time, in view of pro-inflammatory and pro-oxidant^65,66^ effects of heavy metals as well as their contribution to insulin resistance^67^, expanded adipose tissue seems to be dysfunctional, also contributing to aggravated metabolic risk.

Further studies are required to reveal the particular dose- and species-dependent effects of heavy metal exposure on adipogenesis and adipose tissue functioning. Moreover, it is unclear whether other toxic metals, including aluminum, nickel, and beryllium, may also target adipose tissue. These findings could help to unravel the particular role of heavy metal exposure in obesity and metabolic syndrome and the subsequent development of protective strategies.
